# IFN-λ4 Exhibits Differential Induction and Antiviral Activity in RSV and HMPV Infections

**DOI:** 10.3390/v18010111

**Published:** 2026-01-14

**Authors:** Iván Martínez-Espinoza, Pius I. Babawale, Antonieta Guerrero-Plata

**Affiliations:** Department of Pathobiological Sciences, School of Veterinary Medicine, Louisiana State University, Baton Rouge, LA 70803, USA

**Keywords:** RSV, HMPV, interferon, IFN-λ4, respiratory, epithelial cells

## Abstract

Interferons (IFNs) are essential mediators of the innate immune response to viral infections. Among the type III IFNs, the role of IFN-λ4 in respiratory viral infections remains largely understudied. Respiratory syncytial virus (RSV) and human metapneumovirus (HMPV) are clinically significant pneumoviruses that elicit divergent IFN responses in epithelial cells. Here, we investigate the virus-specific induction and antiviral activity of IFN-λ4 by HMPV and RSV infections. We demonstrate that RSV induces a limited expression of IFN-λ4, which is regulated by the expression of the NS1 protein. Furthermore, RSV and HMPV rely primarily on RIG-I for IFN-λ4 induction. Finally, we show that IFN-λ4 exerts antiviral activity against both viruses, with RSV displaying greater sensitivity. These findings highlight the antiviral role of IFN-λ4 to clinically relevant respiratory viruses.

## 1. Introduction

Interferons (IFNs) are pivotal cytokines in the innate antiviral response, categorized broadly into type I (IFN-α/β/ε/ω), type II (IFN-γ), and type III (IFN-λ1/2/3/4) families [1,2,3,4,5,6,7]. In vitro and in vivo studies demonstrate that type I IFNs (IFN-α, IFN-β, IFN-ε) [8,9,10,11,12] and type III IFNs (IFN-λ1, IFN-λ2, IFN-λ3) [12,13,14,15,16] both exert potent antiviral effects against HMPV and RSV, markedly reducing viral replication and spread. Within the type III IFN family, IFN-λ4 is the most recently described member, distinguished by unique genetic regulation, and its expression is determined by a dinucleotide polymorphism that governs protein production [17,18,19,20]. Despite its established impact in hepatotropic infections [21,22,23], the role of IFN-λ4 in respiratory viral disease remains underexplored.

Respiratory syncytial virus (RSV) and human metapneumovirus (HMPV) are closely related pneumoviruses and leading causes of lower respiratory tract infections in infants, the elderly, and immunocompromised patients [24,25,26,27,28,29,30]. Both viruses account for approximately 5–10% of pediatric hospitalizations for acute respiratory infections and present a clinical spectrum [25,26,29,30,31,32,33]. Despite the genetic and structural similarities between HMPV and RSV, these two pneumoviruses elicit markedly different IFN responses, playing a role in the antiviral response [10,34,35]. HMPV and RSV trigger type I IFN responses in airway epithelial cells, but HMPV consistently elicits a stronger overall response, especially in pediatric samples [34]. Specifically, RSV-infected epithelia produce lower levels of IFN-β than those infected with HMPV [13]. Although IFN-α appears earlier after HMPV infection, RSV tends to induce a more pronounced IFN-α peak at later time points. By contrast, IFN-ε expression levels are comparable between HMPV and RSV infections [8,13]. Previous work showed that HMPV infection robustly upregulates IFN-λ1, IFN-λ2, IFN-λ3, and IFN-λ4 in A549 cells. In contrast, RSV induces expression of IFN-λ1–3 to a lesser extent than HMPV and leads to only a marginal increase in IFN-λ4 under comparable multiplicities of infection [13], suggesting that RSV employs selective evasion strategies to suppress IFN-λ4 induction. This virus-specific disparity in IFN-λ4 expression underscores the need to dissect the molecular sensors and viral antagonists governing IFN-λ4 regulation during RSV infection and to define the unique antiviral functions of IFN-λ4 in the respiratory epithelium.

In this study, we characterized the differential induction of IFN-λ4 and defined the contribution of pattern-recognition receptors (PRRs) to the transcription of IFN-λ4 during HMPV and RSV infection. We demonstrate that the NS1 protein from RSV acts as the main antagonist of IFN-λ4 in epithelial cells. More importantly, IFN-λ4 showed antiviral activity against pneumoviruses, revealing that RSV is markedly more susceptible to IFN-λ4 than HMPV. Overall, these findings underscore the relevance of IFN-λ4 in shaping the host response to pneumovirus infection.

## 2. Materials and Methods

### 2.1. Cell Culture

A549 cells were purchased from the American Type Culture Collection (ATCC CCL-185, Manassas, VA, USA). Knockout (KO) A549 cells (RIG-I-KO, MDA-5-KO, and MyD88-KO) were generated by CRISPR-Cas9 as previously described [8]. Cells were grown in F12K medium (Corning, Glendale, AZ, USA) supplemented with 10% fetal bovine serum (FBS) (Gibco, Gaithersburg, MD, USA) and 1% penicillin/streptomycin (Gibco, Gaithersburg, MD, USA) and maintained in a 5% CO_2_ incubator at 37 °C. KO cells were used between passages 6 and 12.

LLC-MK2 cells (ATCC, CCL-7, Manassas, VA, USA) and HEp-2 cells (ATCC, CCL-23, Manassas, VA, USA) were routinely propagated in MEM/EBSS medium (HyClone, Logan, UT, USA) containing 10% FBS (Gibco, Gaithersburg, MD, USA) and 1% penicillin-streptomycin (Gibco, Gaithersburg, MD, USA).

### 2.2. Virus Stocks

The HMPV CAN97-83 strain and the recombinant HMPV encoding green fluorescent protein (HMPV-GFP) were obtained from the Centers for Disease Control and Prevention and ViraTree (ViraTree LLC, Research Triangle Park, NC, USA), respectively, and propagated in LLC-MK2 cells as previously described [10,36]. The RSV A2 strain was obtained from ATCC (Manassas, VA, USA), and the recombinant RSV-A2 expressing red fluorescent protein (rrRSV) was generated as previously described [35]. RSV viruses were propagated in HEp-2 cells. All viruses were purified using a sucrose gradient and titrated by a methylcellulose overlay plaque assay, as previously reported [37,38,39].

### 2.3. Western Blot

Whole cell lysates of A549 cells uninfected and infected with either HMPV or RSV were prepared in 0.5 M Tris-HCl pH 7.5 (Invitrogen, Waltham, MA, USA), 1 M NaCl (Thermo Fisher Scientific, Waltham, MA, USA), 1% Triton X-100 (Thermo Fisher Scientific, Waltham, MA, USA), and complete EDTA (Invitrogen, Waltham, MA, USA), and protease inhibitor cocktail (Millipore Sigma, Hayward, CA, USA). An equal amount of the lysates was loaded onto a reducing SDS-PAGE gel, and following electrophoretic separation, proteins were transferred to polyvinylidene difluoride (PVDF) membranes (Bio-Rad Laboratories Inc., Hercules, CA, USA). Blots were blocked at room temperature for 1 h with 3% BSA in TBST buffer containing 20 mM Tris-base (Signa-Aldrich, St. Louis, MO, USA, 150 mM NaCl, 0.1% Tween (TBST).

Blots were incubated with mouse anti-IFN-λ4 clone # 991620 (R&D Systems, Minneapolis, MN, USA) in 3% BSA in TBST buffer containing 0.1% Tween-20 at room temperature, with gentle agitation for 2 h. Blots were washed three times for 10 min with TBST and then incubated at room temperature with gentle agitation for 2 h with an anti-mouse IgG HRP-conjugated antibody (Cell Signaling Technology, Danvers, MA, USA). For GAPDH, blots were incubated with rabbit anti-GAPDH HRP-conjugated antibody (Cell Signaling Technology, Danvers, MA, USA) at room temperature with gentle agitation for 2 h. Blots were then washed as described and developed using the ECL Plus substrate (Thermo Fisher Scientific, Waltham, MA, USA) and imaged using the Azure Chemiluminescent Western Blot Imager (Azure Biosystems, Dublin, CA, USA).

### 2.4. Agarose Gel Electrophoresis

Product of RT-qPCR targeting IFN-*λ*4, IFN-*λ*2/3, IFN-β, and GAPDH was resolved on a 1% agarose gel prepared in 1X Sodium Borate. Samples were mixed with 6X loading dye and run alongside a 100-base pair DNA ladder (Bio-Rad Laboratories Inc., Hercules, CA, USA). Electrophoresis was performed at 100 V for 50 min. Gels were imaged using the Azure imager (Azure Biosystems, Dublin, CA, USA).

### 2.5. Generation of RSV-NS1 and NS2 Knockdown (KD) A549 Cells

Stable cell lines constitutively expressing shRNA targeting the non-structural (NS) proteins NS1 and NS2 of RSV were generated using the pLKO-TCR lentiviral vector (Addgene, Watertown, MA, USA). Gene-specific shRNA sequences were designed for each target and cloned into the pLKO-TCR vector using the EcoRI and AgeI restriction sites. For lentivirus production, 293FT cells were co-transfected with the shRNA-containing pLKO-TCR construct, psPAX, and pVSV-G using Lipofectamine™ 3000 (Invitrogen, Waltham, MA, USA). Lentiviral particles were harvested 48 h post-transfection and used for transducing A549 cells in the presence of 10 µg/mL polybrene (Millipore Sigma, Hayward, CA, USA) to enhance transduction efficiency. After transduction, cells were selected with 10 µg/mL puromycin (Gibco, Gaithersburg, MD, USA). Surviving clones were subsequently expanded and validated to confirm the generation of NS1-KD and NS2-KD knockdown.

### 2.6. Viral Infection and IFN-λ4 Treatment

Cells were infected with HMPV at a multiplicity of infection (MOI) of 1.0 in the presence of 1 µg/mL trypsin or infected with RSV in serum-free MEM. After 2 h of adsorption, the inoculum was removed and replaced with complete medium, as previously reported [8]. For the experiments testing IFN-λ4 antiviral activity, A549 monolayers were treated with human recombinant IFN-λ4 (R&D Systems, Minneapolis, MN, USA) or BSA at concentrations of 0.2, 0.5, and 1 μg/mL 24 h before viral infection and after removing the viral inoculum. After 24 h of infection, cell lysates were harvested for subsequent analyses.

### 2.7. Cytotoxicity Assay

Cellular cytotoxicity was assessed by measuring lactate dehydrogenase (LDH) release into culture supernatants using the CyQUANT™ LDH Cytotoxicity Assay (ThermoFisher Scientific, Waltham, MA, USA). A549 cells were treated with increasing concentrations of IFN-λ4. Controls included culture medium as a background control and A549 cells treated with lysis buffer as a maximum LDH control. At 24 h post-treatment, cell-free supernatants were tested for LDH content according to the manufacturer’s instructions. Absorbance was recorded at 490 nm with a reference wavelength of 680 nm using a microplate reader. Percent cytotoxicity was calculated by normalizing sample LDH release to the maximum LDH control and subtracting background from the culture medium.

### 2.8. RNA Extraction and Quantitative Real-Time Reverse Transcription-PCR (RT-qPCR)

Total RNA was extracted using the RNeasy Plus Kit (Qiagen, Germantown, MD, USA). cDNA was synthesized with the LunaScript RT SuperMix Kit (New England Biolabs, Ipswich, MA, USA), and gene expression was quantified using PowerTrack SYBR Green Master Mix (Thermo Fisher Scientific, Waltham, MA, USA). qPCR was performed on a QuantStudio™ 12K PCR system (Applied Biosystems, Foster City, CA, USA) with primers targeting *NS1, NS2, IFN-β, IFN-λ2/3, IFN-λ4, ISG56/IFIT1, ISG54/IFIT2, ISG60/IFIT3, ISG15, MX1*, and *GAPDH* (IDT, Coralville, IA, USA) ). Relative expression was calculated using the ΔΔCT method with GAPDH as the internal control. Viral copy numbers contained in 1 ng/μL of total RNA (Viral copies/ng) were determined by absolute quantification using standard curves from plasmids containing HMPV or RSV N genes. Data were analyzed using QuantStudio™ 12K Flex Software v1.3 (Applied Biosystems, Foster City, CA, USA).

### 2.9. Live-Cell Imaging and Analysis

Cells infected with HMPV-GFP or rrRSV, with or without rhIFN-λ4 treatment, were monitored using an Incucyte^®^ Zoom HD/2CLR time-lapse microscope (Sartorius, Bohemia, NY, USA). Images were captured at an exposure of 400 ms for the green (GFP) channel and 800 ms for the red channel. Fluorescence was quantified as Green Calibrated Units (GCU) or Red Calibrated Units (RCU) per µm^2^ per image, then normalized to the signal from untreated infected cells and expressed as a percentage.

### 2.10. Statistical Analysis

Statistical analyses were calculated by an unpaired *t*-test to compare two normally distributed groups. One-way analysis of variance (ANOVA) followed by the appropriate post hoc tests to correct for multiple comparisons was used to determine the differences between three or more groups. Two-way ANOVA with Tukey’s multiple comparisons post hoc test was used to evaluate two categorical variables (Infection) × (Condition of knock down genes). The results are expressed as mean ± standard errors of the mean (SEM). A *p*-value of 0.05 or lower was considered significant. GraphPad Prism 10.6.1 (GraphPad Software, San Diego, CA, USA) was used for statistical analyses.

## 3. Results

### 3.1. Differential Expression of IFN-λ4 by RSV and HMPV

**Figure 1:**
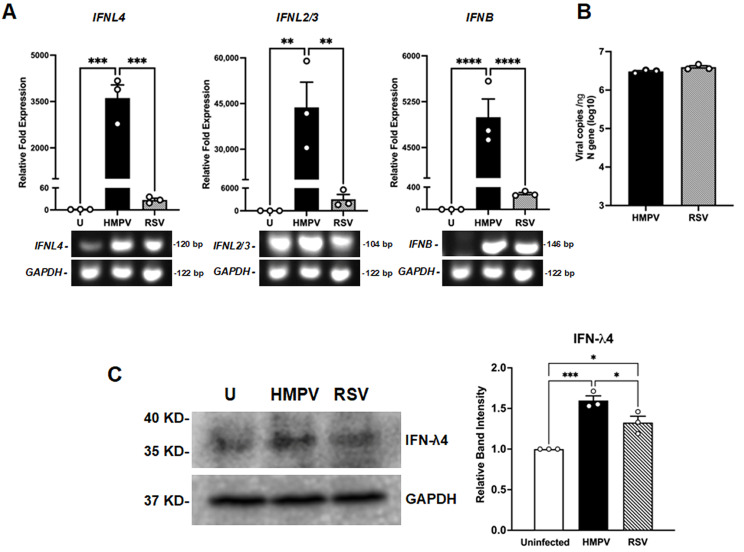
Induction of IFN-λ4 in epithelial cells infected with HMPV and RSV. A549 cells were infected with HMPV or RSV at an MOI of 1.0 for 24 h. (**A**) Gene expression of IFNL4 (IFN-λ4), IFNL2/3 (IFN-λ2/3), and IFNB (IFN-β) was quantified by RT-qPCR. PCR products were run on a 1% agarose gel. (**B**) Viral copies/ng of RNA for the N gene of HMPV or RSV were determined by absolute quantification. (**C**) Cell lysates from HMPV- or RSV-infected cells were collected for Western blot analysis to detect IFN-λ4. Relative band intensity was calculated using the endogenous housekeeping protein GAPDH. Bars represent the mean ± SEM from three independent experiments. Statistical differences were assessed using a one-way ANOVA followed by Tukey’s multiple comparison test (**A**,**C**) and Student’s *t*-test (**B**). * *p* < 0.05, ** *p* < 0.01, *** *p* < 0.001, **** *p* < 0.0001.

A549 epithelial cells were infected at a multiplicity of infection (MOI) of 1.0 with HMPV or RSV for 24 h, followed by total RNA extraction. The expression of IFN-λ4 transcript levels was quantified by RT-qPCR. For comparison, the expression of IFN-λ2/3 and IFN-β was also analyzed. As shown in Figure 1A, HMPV induced a markedly higher IFN-λ4 response with a 3612 ± 425-fold increase compared to RSV, which showed a 27 ± 4.8-fold increase. To determine whether this differential pattern extended to other members of the type III IFN family, we assessed the expression of IFN-λ2/3, which represents the most abundantly expressed and biologically active isoforms in mucosal antiviral response [40,41]. We found that HMPV also strongly upregulated IFN-λ2/3 expression with a 43,720-fold increase, whereas RSV elicited a significantly lower response of 3028-fold (Figure 1A). We also examined the induction of IFN-β, a key member of the type I IFN family involved in antiviral defense. As observed, HMPV induced a markedly higher expression of IFN-β (4996 ± 289-fold) compared to RSV (280.2 ± 24.4-fold). To rule out the possibility that differences in host IFN responses were due to variations in viral infection levels, we quantified the viral replication in infected cells by absolute quantification of the viral N gene by RT-qPCR. Data shown in Figure 1B indicate that HMPV and RSV replicated to comparable levels in our experimental conditions (6.48 and 6.59 log_10_ copies/ng for HMPV and RSV, respectively), suggesting that the observed differences in IFN induction cannot be attributed to disparities in viral load.

Furthermore, Western blot assays and quantitative densitometry analysis indicated that HMPV also induced higher levels of IFN-λ4 than RSV at the protein level (Figure 1C). Together, these data reveal that HMPV elicits a more robust induction of IFN-λ4 than RSV in epithelial cells. However, IFN-λ4 was less induced by HMPV compared to IFN-λ2/3, under the same conditions.

### 3.2. Contribution of RIG-I, MDA5, and MyD88 on the Induction of IFN-λ4 by RSV and HMPV

**Figure 2:**
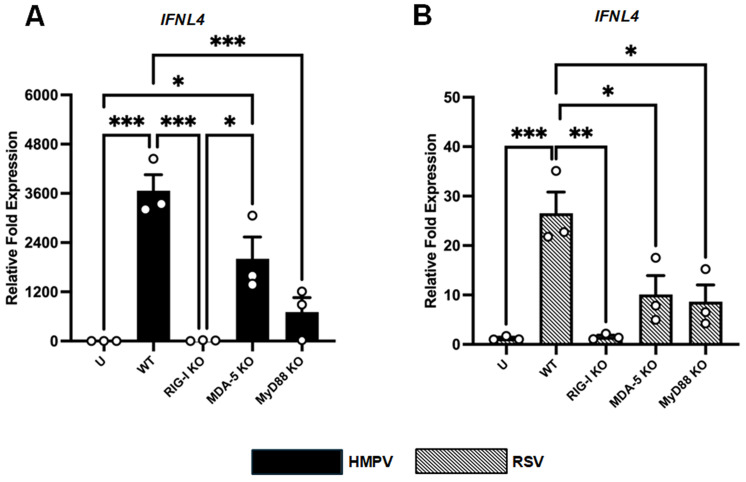
Contribution of RIG-I, MDA5, and MyD88 in the induction of IFN-λ4 by RSV and HMPV in A549 cells. WT cells and cells knocked out for RIG-I (RIG-I-KO), MDA-5 (MDA5-KO), and MyD88 (MyD88-KO) were infected with (**A**) HMPV or (**B**) RSV at an MOI of 1.0 for 24 h. Cell lysates were collected and analyzed for gene expression of IFN-λ4 by RT-qPCR. Bars show mean ± SEM from three independent experiments. Statistical differences were calculated using one-way ANOVA followed by a Sidak’s multiple comparison test. * *p* < 0.05, ** *p* < 0.01, *** *p* < 0.001. U = Uninfected.

Pattern-recognition receptors (PRRs) constitute the frontline sensors of viral infection, detecting pathogen-associated molecular patterns via cytosolic receptors such as retinoic acid-inducible gene-I (RIG-I) and melanoma differentiation-associated gene 5 (MDA5) or endosomal Toll-like receptors (TLRs) that signal through the adaptor myeloid differentiation primary response 88 (MyD88) [42]. Upon sensing the viral infection, the IFN regulatory factors (IRFs) are activated, leading to the induction of the IFN response [1,43,44]. To delineate which PRRs govern IFN-λ4 induction by HMPV and RSV, we used A549 epithelial knockout (KO) cell lines lacking RIG-I, MDA5, or MyD88, as we previously validated [8]. The wild type (WT) and KO cells were infected with HMPV or RSV at an MOI of 1.0 for 24 h. After that time, cell lysates were collected for RT-qPCR analysis. Our results indicate that, in the case of HMPV, the deletion of RIG-I significantly reduced the IFN-λ4 expression compared to WT cells. Similarly, knocking out MyD88 markedly decreased expression IFN-λ4. However, compared to WT cells, the lack of MDA5 reduced the induction of IFN-λ4 by 45%, but this difference was not statistically significant (Figure 2A). Regarding the induction of IFN-λ4 during RSV infection, we observed that IFN-λ4 was substantially reduced in the absence of RIG-I. A similar effect was observed in cells lacking MDA5 and MyD88, resulting in a significant reduction of IFN-λ4 expression. Together, these data demonstrate that RSV and HMPV induce IFN-λ4 through different mechanisms, where RSV relies on a combination of cytosolic and endosomal sensing pathways to regulate IFN-λ4 expression, and HMPV primarily depends on RIG-I and MyD88, with minimal involvement of MDA5.

### 3.3. Effect of RSV NS1 and NS2 on the Expression of IFN-λ4

**Figure 3:**
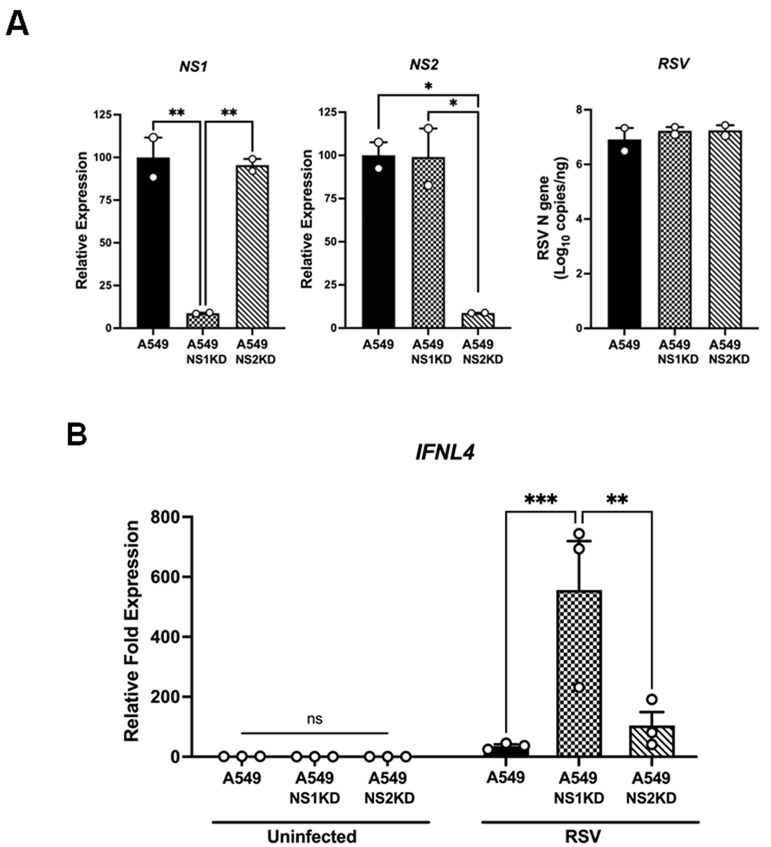
Role of RSV NS1 and NS2 proteins in IFN-λ4 induction. A549 cells and stable A549 cell lines constitutively expressing shRNA to knock down NS1 (NS1KD) or NS2 (NS2KD) were infected with RSV at an MOI of 1.0 for 24 h and analyzed for: (**A**) expression of NS1, NS2, and viral copy numbers of the N gene; and (**B**) relative fold expression of IFNL4 (IFN-λ4) by RT-qPCR. Bars show mean ± SEM from three independent experiments. Statistical differences were calculated using one-way and two-way ANOVA followed by Tukey’s multiple comparison test. ns = not significant; * *p* < 0.05, ** *p* < 0.01, *** *p* < 0.001.

Next, we explored the mechanisms by which RSV induced a poor IFN-λ4 response compared to HMPV. One of the main genomic differences between these two viruses is the presence of two nonstructural (NS) proteins in RSV, NS1 and NS2, which are absent in HMPV. These proteins are known to antagonize type I IFN production and response in epithelial cells and macrophages [45,46,47,48,49]. Thus, we hypothesized that NS1 and/or NS2 could also be responsible for the observed suppression of the IFN-λ4 expression by RSV. To investigate the contribution of RSV NS proteins, we generated knockdown A549 cells that constitutively interfered with the expression of NS1 or NS2 proteins (NS1-KD and NS2-KD). To validate the knockdown efficiency of the NS genes, A549 cells and the NS1-KD and NS2-KD were infected with RSV at an MOI of 1.0 for 24 h, and the expression levels of NS1 and NS2 transcripts were then quantified by RT-qPCR. As shown in Figure 3A, the transcript levels of NS1 and NS2 were reduced by ~91% compared to A549 cells; however, the RSV replication was comparable in all three groups of cells, validating the successful and selective silencing of the RSV NS1 and NS2 genes in the respective KD cell lines.

Following validation of knockdown efficiency, cells were infected with RSV at an MOI of 1.0 for 24 h, and IFN-λ4 transcripts were determined by RT-qPCR analysis. Data shown in Figure 3B indicate that, when compared to A549 cells, knocking down NS1 substantially increased IFN-λ4 expression after RSV infection, with transcript levels increasing from 35 ± 32 fold to 556.36 ± 31 fold. However, NS2-KD led to a lower increase of 104.12 ± 45-fold. Overall, these data suggest that although both NS1 and NS2 have an inhibitory effect on the expression of IFN-λ4, NS1 has a major antagonist role.

### 3.4. Antiviral Response Induced by IFN-λ4

**Figure 4:**
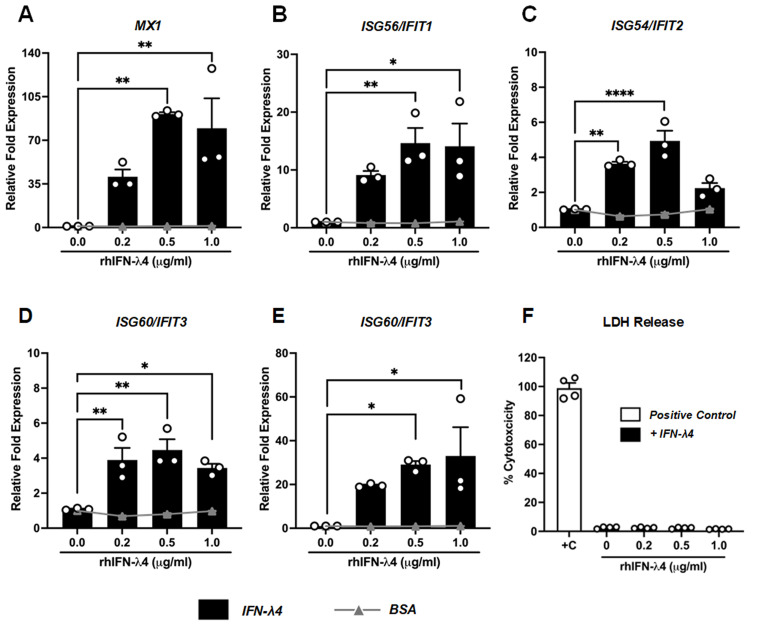
Induction of ISGs and cytotoxicity in A549 cells. Cells were treated with increased concentrations of rhIFN-λ4 or BSA for 24 h (0.2, 0.5, and 1 μg/mL). Relative fold expression of (**A**) *MX1*, (**B**) *ISG56/IFIT1*, (**C**) *ISG54*/*IFIT2*, (**D**) *ISG60*/*IFIT3* and (**E**) *ISG15* was determined by RT-qPCR. (**F**) The percentage of cytotoxicity was determined by LDH release. Bars and lines show mean ± SEM from three independent experiments (*n* = 3). Statistical differences were calculated using one-way ANOVA followed by a Dunnett’s multiple comparison test. * *p* < 0.05, ** *p* < 0.01, **** *p* < 0.0001.

Type III IFNs are key mediators of the innate immune response. Once secreted, these cytokines bind to their respective receptors on both infected and neighboring cells, initiating antiviral signaling cascades through a heterodimeric receptor complex composed of IFNLR1 and IL-10R2, which is primarily expressed on epithelial cells [21]. This engagement activates downstream signaling pathways that drive the expression of a broad array of IFN-stimulated genes (ISGs), collectively establishing an antiviral state to suppress viral replication and shape the host immune response [4]. Here, we first characterized the ISG response induced by rhIFN-λ4 at different concentrations, ranging from 0.2 to 1.0 μg/mL. We measured the expression of five ISG genes (*MX1*, *IFIT1*, *IFIT2*, *IFIT3*, and *ISG15*), each chosen for its distinct mechanism of viral restriction. Our results indicate that *MX1*, a dynamin-like GTPase that blocks viral nucleocapsid import [50], was robustly induced from 40.7 ± 5.8-fold at 0.2 µg/mL to 91.1 ± 1.4-fold at 0.5 µg/mL, and remained high (79.63 ± 23.99-fold) at 1 µg/mL of rhIFN-λ4 (Figure 4A). *ISG56/IFIT1*, which binds uncapped 5′-triphosphate RNA viral RNAs to block their translation [51], increased from 9.1 ± 0.7-fold at 0.2 µg/mL to 14.6 ± 2.6-fold at 0.5 µg/mL and remained elevated at 1 µg/mL (14.09 ± 3.93-fold) (Figure 4B). *ISG54/IFIT2*, known to promote apoptosis of infected cells and limit viral spread [52], was slightly induced from 3.6 ± 0.1-fold at 0.2 µg/mL to 4.9 ± 0.6-fold at 0.5 µg/mL before settling back to 2.2 ± 0.3-fold at 1 µg/mL (Figure 4C). Similarly, *ISG60/IFIT3,* a scaffold protein that amplifies IFIT1/2 signaling [53], was modestly increased from 3.9 ± 0.7 to 4.5 ± 0.6-fold at concentrations of 0.2 and 0.5 µg/mL, respectively, and remained in 3.4 ± 0.2-fold at 1 µg/mL (Figure 4D). Finally, *ISG15*, which conjugates to viral and host proteins to disrupt multiple stages of the viral life cycle [54], was induced from 19.5 ± 0.4-fold at 0.2 µg/mL to 33.0 ± 13.1-fold at 1 µg/mL (Figure 4E). To evaluate the effect of rhIFNL4 on cell viability, we assessed the percentage of cytotoxicity by measuring lactate dehydrogenase (LDH) release in response to rhIFNL4. The percentage of cytotoxicity was relative to the positive control (+C) from lysed cells. Data in Figure 4F show that the cytotoxicity observed was low, ranging from 2.61% in untreated cells to a maximum of 2.43% in the cells treated with rhIFN-λ4. Together, these data demonstrate that rhIFN-λ4 induces low cytotoxicity with a broad, dose-dependent ISG network, with each component targeting distinct aspects of the viral replication cycle, thereby establishing its ability to activate the antiviral programs required to restrict viral replication. Notably, *MX1* was among the most highly induced ISGs following rhIFN-λ4 treatment. As a control of specificity, the effect of BSA was included at the same concentrations as rhIFN-λ4. Data shown in Figure 4A–E (grey lines) indicated that BSA did not induce the expression of ISGs, validating the specificity of the effect of IFN-λ4 in inducing an antiviral state in the treated cells.

### 3.5. Antiviral Effect of IFN-λ4 to RSV and HMPV Infection

**Figure 5:**
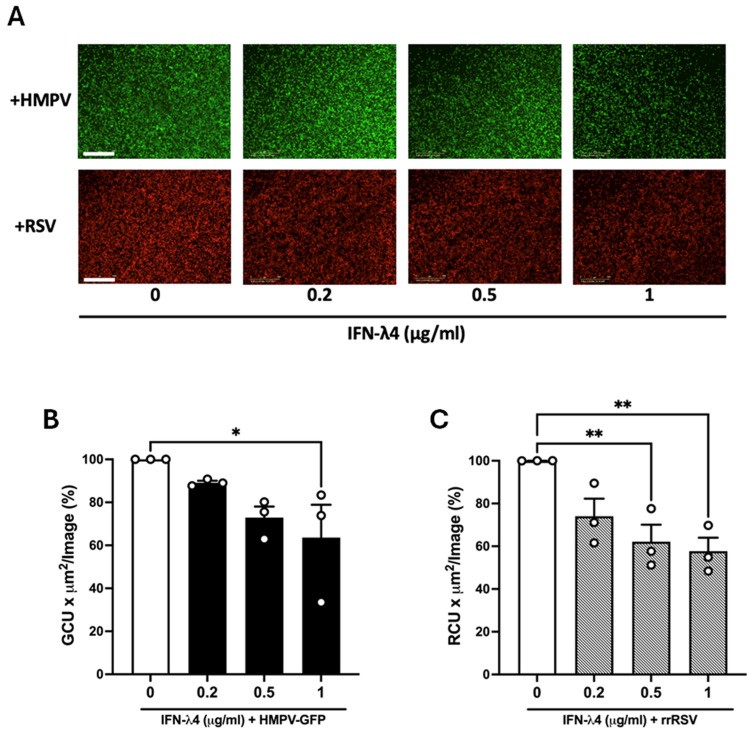
Susceptibility of RSV and HMPV infection to IFN-λ4. Cells were treated with rhIFN-λ4 for 24 h at concentrations of 0.2, 0.5, and 1.0 μg/mL, followed by infection with RSV (rrRSV) or HMPV (HMPV-GFP) at an MOI of 1.0 for 24 h. Viral susceptibility was determined by the detection of fluorescence signal using the IncuCyte^®^ system. (**A**) Representative images of cells infected with rrRSV or HMPV-GFP. Scale bar: 800 μm. (**B**,**C**) Fluorescence intensity is presented as a percentage normalized to untreated cells, which were set to 100%, showing the reduction observed in cells infected with (**B**) HMPV or (**C**) RSV. Bars show mean ± SEM from three independent experiments (*n* = 3). Statistical differences were calculated using one-way ANOVA followed by a Dunnett’s multiple comparison test. * *p* < 0.05, ** *p* < 0.01.

Based on the observed antiviral response induced by IFN-λ4, we sought to determine its antiviral effect on RSV and HMPV infection. A549 cells were pretreated with rhIFN-λ4 for 24 h, followed by infection with either HMPV-GFP or rrRSV at an MOI of 1.0. We first determined the susceptibility of RSV and HMPV to IFN-λ4 by defining the percentage of infected cells by live-cell imaging. Because the reporter expression of HMPV-GFP or rrRSV is dependent on viral replication, these recombinant systems provide a robust and quantitative approach to evaluate the level of infectivity in IFN-λ4-treated cells. The data shown in Figure 5A revealed a dose-dependent reduction in the fluorescent signal for both viruses in the presence of IFN-λ4. Moreover, the quantification of the signal indicated that HMPV showed a modest decrease in infectivity at 0.2 and 0.5 µg/mL, but a significant reduction of ~36% at 1 µg/mL (Figure 5B). In comparison, RSV infectivity significantly decreased the percentage of RSV-infected cells by 38% at a concentration of 0.5, and by 42% at 1 µg/mL (Figure 5C). These data suggest that rhIFN-λ4 confers a dose-dependent antiviral activity, effectively limiting viral infection, where RSV exhibited a greater degree of sensitivity to rhIFN-λ4 than HMPV.

**Figure 6:**
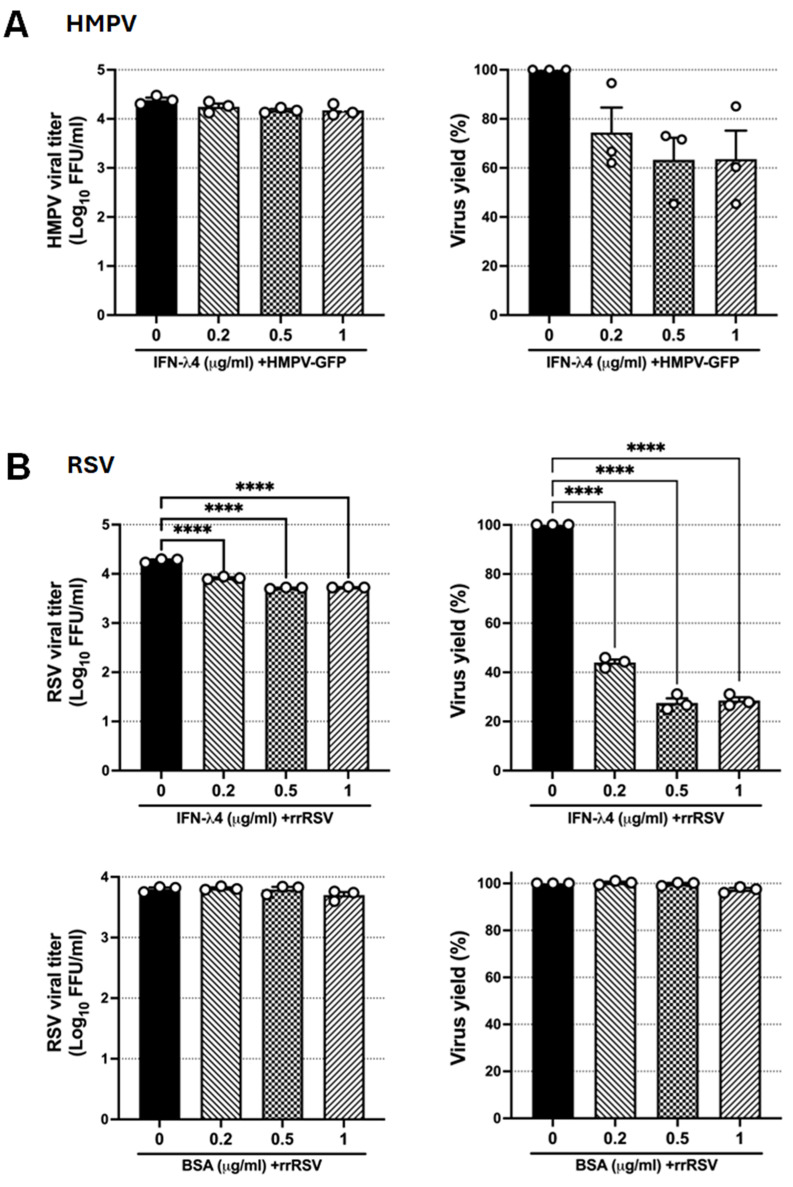
Effect of IFN-λ4 on RSV and HMPV replication. Cells were pretreated for 24 h with increasing concentrations of rhIFN-λ4. BSA was included as a control of specificity. Cells were then infected with rrRSV or HMPV-GFP at an MOI of 1.0 for 24 h. Viral titers in cell-free supernatants were quantified by plaque assay and expressed as log10 PFU/mL or as percentage of virus yield relative to untreated controls for (**A**) HMPV and (**B**) RSV. Bars show mean ± SEM from three independent experiments (*n* = 3). Statistical differences were calculated using one-way ANOVA followed by a Dunnett’s multiple comparison test. **** *p* < 0.0001.

To further assess the antiviral effect of IFN-λ4, we determined the release of virions. Epithelial cells were pretreated with rhIFN-λ4 and subsequently infected 24 h later with either HMPV-GFP or rrRSV. Supernatants were collected after 24 h of infection, and viral titers were determined by plaque assay. Figure 6A shows that rhIFN-λ4 restricted HMPV titers, although with a marginal reduction of 0.14 log_10_ and ~30% viral yield (Figure 6A). In contrast, RSV titers were significantly decreased by 0.4 log_10_ FFU/mL, representing approximately a 57% reduction in viral yield when a concentration of 0.2 µg/mL was used. Furthermore, concentrations of 0.5 and 1 µg/mL reduced the replication of RSV by 0.6 log_10_ FFU/mL with a viral yield of ~75% (Figure 6B, upper panels). To confirm the specificity of the antiviral effect of rhIFN-λ4 on RSV replication, cells were pretreated with BSA at the same concentrations as the antiviral cytokine. Data shown in Figure 6B (lower panels) indicate that no antiviral effect was induced by BSA. Overall, these results demonstrate that rhIFN-λ4 effectively establishes an antiviral state in epithelial cells, with RSV displaying greater sensitivity than HMPV in both viral spread and release of infectious particles.

## 4. Discussion

Type III IFNs (IFN-λ1, λ2, λ3, and λ4) are key components of the innate antiviral immune response that signal through a receptor complex composed of IFNLR1 and IL10R2 [40]. Expression of IFNLR1 is largely restricted to epithelial cells at mucosal surfaces, including the respiratory tract. As a result, type III IFNs induce a localized antiviral state at barrier tissues with limited systemic inflammation [40,55,56,57]. This compartmentalization allows type III IFNs to provide frontline mucosal protection while minimizing immune-mediated tissue damage [18,40,57,58,59]. They induce a core set of ISGs that inhibit virus entry, replication, and spread at barrier sites [60]. Among these, IFN-λ4 is distinguished by unique genetic regulation, and its expression is governed by a common dinucleotide polymorphism [21,61]. Although IFN-λ4 is less well characterized in respiratory infections, emerging evidence of the antiviral activity of the type III IFN family against other viruses suggests IFN-λ4 contributes critically to controlling pathogens at the airway epithelium [1,19,20,62,63,64,65,66,67,68].

We have previously reported that HMPV triggers a more robust expression of IFN response than RSV in A549 cells [13]. Here, we confirmed that, compared to HMPV, RSV induces a reduced expression of IFN-λ4 (Figure 1), suggesting a distinct mechanism of IFN-λ4 induction by these viruses. PRRs are essential for detecting viral invasion and initiating IFN responses [42]. We found that the IFN-λ4 induction was strongly dependent on RIG-I expression during RSV infection, revealing the importance of 5′-triphosphate–bearing short dsRNA generated during viral infection and detected by this sensor. MDA5 was also necessary for the induction of IFN-λ4 by RSV, suggesting that longer dsRNA structures contribute to activating MDA5 and triggering IFN-λ4 expression [69,70,71]. Moreover, RSV also relied on the expression of MyD88 to induce IFN-λ4, implicating recognition by TLRs signaling via MyD88, such as TLR7 and TLR8, which recognize ssRNAs [72,73,74,75,76]. During HMPV infection, IFN-λ4 induction was almost entirely abrogated in RIG-I KO cells, and the absence of MyD88 also led to a significant decrease. However, the deletion of MDA5 had no significant effect, indicating that HMPV relies primarily on RIG-I and endosomal TLRs to drive IFN-λ4 expression. These findings reveal the considerable contribution of RIG-I for IFN-λ4 induction in epithelial cells by RSV and HMPV.

The limited induction of IFN-λ4 by RSV suggested a viral antagonist mechanism. Given that RSV NS1 and NS2 are established inhibitors of the IFN response [47,49,77,78], we assessed their roles in IFN-λ4 regulation (Figure 3). Our data indicate that NS1 is the main suppressor of IFN-λ4; however, NS2 also contributes to its regulation (Figure 3B). These findings are similar to those reporting the role of NS1 and NS2 in the induction of IFN-λ1 and IFN-λ2/3 using recombinant RSV viruses lacking NS1 and NS2 proteins, where ΔNS1 was identified as the primary antagonist, followed by ΔNS2 [45]. Mechanistically, RSV inhibits both type I and type III interferon responses primarily through the coordinated actions of its NS proteins. At the level of IFN induction, NS2 binds and antagonizes RIG-I, blocking RIG-I–MAVS signaling and IFN-β promoter activation [77]; NS1 also targets the upstream pathway by binding TRIM25 and preventing the K63-linked ubiquitination of RIG-I required for signaling [48]. Functionally, recombinant viruses lacking NS1 or NS2 show enhanced type I/III IFN responses, underscoring that NS1/NS2 suppress both IFN classes in infected cells [45]. Consistent with these findings, our data identify RIG-I as a key PRR for IFN-λ4 induction, suggesting that NS1/NS2 may target RIG-I to blunt IFN-λ4 induction.

Treatment of A549 cells with IFN-λ4 induced no more than 2.5% cell death at any concentration tested, demonstrating minimal cytotoxicity. This favorable safety profile aligns with reports on the cytotoxic effect of IFN-λ, indicating that no measurable cytotoxicity by rhIFN-λ1 and IFN-λ2 was observed in corneal epithelial cells [79]. Moreover, IFN-λ3 preserved cell viability in HepG2.2.15 cells [80].

The further induction of ISGs by rhIFN-λ4 suggests that IFN-λ4 induces an antiviral state in A549 cells as demonstrated by the expression of *MX1*, *IFIT1*, *IFIT2*, *IFIT3*, and *ISG15* (Figure 4), each targeting distinct stages of viral replication [50,51,52,53,54,81,82]. We showed that *MX1* exhibited the highest induction. *MX1* is a gene encoding MxA [50,83,84], a dynamin-like GTPase induced by IFNs, which assembles into oligomeric ring-like structures through GTP-driven conformational changes [85]. These oligomers encircle incoming viral ribonucleoprotein (RNP) complexes, selectively targeting them to prevent uncoating, block cytoplasmic trafficking, or interfere with nuclear import. This mechanism effectively suppresses transcription and replication of a broad spectrum of RNA viruses [86]. *MX1*-mediated restriction is particularly potent against orthomyxoviruses, such as influenza, where it inhibits nucleocapsid nuclear translocation. It also restricts bunyaviruses and RSV by similar mechanisms [68,85,86]. Our results demonstrate the strong induction of *MX1* and are consistent with findings reported for other type III IFNs. For example, treatment of HepG2.2.15 cells with IFN-λ3 led to a ~150-fold increase in *MX1* expression [80]. In another comparative study of IFN-λ3 and IFN-λ4, similar biological activities were observed in their ability to induce *MX1* expression [87]. IFN-λ4 also induced the expression of *ISG15,* and to a lesser extent, *IFIT1*, *IFIT2*, and *IFIT3* (Figure 4). ISG15 can curb RNA virus replication through ISGylation, a covalent modification carried out by the E1 enzyme UBE1L, E2 UBE2L6/UBCH8, and E3 ligases such as HERC5 and TRIM25, with the de-ISGylase USP18 removing ISG15 from substrates [54,88]. In fact, it has been reported that in RSV-infected airway cells, ISG15 exhibits an antiviral effect by reducing RSV replication via an ISGylation-dependent mechanism [82]. The susceptibility of RSV to IFITs has also been explored using genetic knockout and ectopic overexpression of IFIT1–3. It has been shown that modulation of RSV replication in vitro, loss of IFITs enhances viral growth, whereas their overexpression suppresses it, demonstrating a direct antiviral role for these proteins [89]. IFN-induced proteins with tetratricopeptide repeats (IFIT1–3) are prominent antiviral effectors known to bind non-self-viral RNAs (such as those lacking 2′-O methylation or with 5′-triphosphate ends), sequester them, or impede translation initiation via interactions with eIF3, forming heteromeric IFIT1/IFIT2/IFIT3 complexes that enhance antiviral potency [51,52,53].

RSV and HMPV were susceptible to IFN-λ4 treatment, demonstrating that IFN-λ4 confers antiviral protection against both pneumoviruses, but is markedly more effective against RSV. Similar antiviral effects have been observed for the hepatitis C virus, coronaviruses, and influenza [21,90,91]. However, to the best of our knowledge, this is the first report on the antiviral effect of IFN-λ4 against respiratory pneumovirus infection. We consider that although recombinant IFN-λ4 can robustly induce ISGs in epithelial cells, HMPV remains weakly inhibited, potentially because the virus can antagonize both the IFN induction and downstream signaling, limiting the execution of the ISG program [92,93,94,95,96,97,98,99]. In particular, the protein SH from HMPV inhibits pSTAT1 and promotes the degradation of JAK1 [92,93,97,98]. Moreover, M2-2 interferes with the RIG-I/TRIM25–MAVS pathway [94,95,96,99], and G dampens the TLR4, RIG-I/MDA5-IRF7 pathways [16,17,18]. Together, these mechanisms curb upstream IFN production and blunt JAK/STAT output, likely reducing ISG protein accumulation. Moreover, Type I IFNs (α/β/ε) exhibit antiviral activity against both HMPV and RSV; however, HMPV is more sensitive to the treatment with type I IFN [8,10,12].

The antiviral potency of IFN-λ4 is broadly comparable to other type III IFNs (e.g., IFN-λ3) in hepatocytes. In contrast, type I IFNs (e.g., IFN-α) often drive a somewhat stronger/wider ISG response in the same assays. For example, recombinant IFN-λ4 signaled via IFNLR1/IL10R2 and showed antiviral activity similar to IFN-λ3, while IFN-α induced slightly higher ISG levels in parallel readouts [21]. In primary and liver-derived cell systems, IFN-λ4 and IFN-λ3 were reported to have comparable antiviral effects, with the caveat that IFN-λ4 secretion/biogenesis can be less efficient, affecting apparent potency outside controlled expression systems [87]. Regarding the ISG program, multiple datasets support the notion that IFN-λ4 induces the canonical type III IFN gene set—i.e., it is not uniquely selective, but somewhat differs mostly in magnitude and kinetics relative to IFN-λ3/IFN-α [21,100].

The viral susceptibility to IFN-λ4 has been previously demonstrated in vitro, mostly from studies of hepatitis C virus (HCV), where recombinant IFN-λ4 restricted viral replication in hepatocyte-derived Huh-7 models and induced a robust ISG response [21]. In the same study, IFN-λ4 also inhibited replication of human coronaviruses, including human coronavirus 229E (HCoV-229E) and Middle East respiratory syndrome-related coronavirus (MERS-CoV), in primary human airway epithelial cultures, demonstrating that its antiviral activity extends beyond hepatotropic viruses [21]. Using recombinant IFN-λ4 variants, Bamford and colleagues further demonstrated that IFN-λ4 suppresses Zika virus (ZIKV), influenza A virus (IAV), and Encephalomyocarditis virus (EMCV) in human epithelial cells, with specific IFN-λ4 polymorphic variants displaying even stronger antiviral potency than ordinary human IFN-λ4 [101]. More recently, Guo et al. confirmed the antiviral activity of IFN-λ4 against EMCV and vesicular stomatitis virus (VSV) in HepaRG and T84 epithelial cell systems, demonstrating that IFN-λ4 induces distinctive, rapid antiviral signaling kinetics [102]. Together, these in vitro models reveal that IFN-λ4 is a broadly acting antiviral cytokine capable of inhibiting diverse RNA viruses across multiple human cell types. However, the effect of IFN-λ4 in vivo against HCV remains controversial as it has a paradoxical effect. Human genetic studies consistently show that individuals who produce functional IFN-λ4 have reduced spontaneous clearance of HCV [103], suggesting that IFN-λ4 signaling may impair antiviral resolution despite its potent antiviral activity in vitro. This paradox has been attributed to the sustained induction of ISGs and desensitization of downstream IFN pathways [104]. However, the recent demonstration that adenoviral delivery of human IFN-λ4 can inhibit IAV replication in mice adds a new dimension to the in vivo effect of IFN-λ4, showing that IFN-λ4 is capable of generating a protective antiviral state against different strains of IAV when expressed directly in the respiratory tract [91]. Together, these findings suggest that the in vivo effects of IFN-λ4 are highly context- and virus-dependent, highlighting the relevance of studying its impact in vivo on other respiratory viral infections. Given the antiviral effect of IFN-λ4 against RSV shown in vitro, future work is warranted to investigate the in vivo effect of IFN-λ4 against RSV infection.

## 5. Conclusions

Overall, our observations demonstrate that RSV and HMPV differentially activate cytosolic receptors to induce the expression of IFN-λ4 and provide evidence that NS1 is a dominant antagonist of IFN-λ4 during RSV infection. More importantly, we demonstrate the antiviral activity of IFN-λ4 against pneumoviruses. Thus, our findings support the contribution of IFN-λ4 in mucosal host defense and highlight its potential relevance to protect against RSV infection.

## Data Availability

The data supporting the conclusions of this research manuscript are all present within the article.
